# Local Dental Societies

**Published:** 1862-10

**Authors:** 


### LOCAL DENTAL SOCIETIES.

In view of the fact that the formation and encouragement of local associations is one prominent object of the "American Dental Association," that body at its recent meeting appointed a committee on local dental societies. The legitimate work of this committee, as the agent of the National Association, will involve the following particulars, viz: To ascertain the condition of all the existing local societies, at least those entitled to representation in this body; the number of active members there are in each; when they were organized; how often they meet; and whether they are in a growing or declining condition, and if the latter, from what cause.

It is also the duty of this committee to encourage and coöperate in the formation of new societies, wherever it is feasible, and an opportunity may afford. An accurate account of all these things should be kept, and from them a report made to the parent society. By this method the present condition of the profession would be most fully brought out.

The committee consists of Dr. W. H. Atkinson, of New York city, and Dr. J. Taft, of Cincinnati, O. The committee will be pleased to receive information from any member of the profession in reference to any of these points. The committee would also with much pleasure confer with any members of the profession in regard to the organization of new societies; and any aid that can be afforded will be cheerfully given. T.

